# Prevalence of psoriatic arthritis among patients with plaque psoriasis: a Brazilian retrospective study

**DOI:** 10.1590/1516-3180.2020.0629.16032021

**Published:** 2021-08-09

**Authors:** Shirley Braga Lima Gamonal, Aloisio Carlos Couri Gamonal, Marcos Antônio Fernandes Brandão, Laura Andrade Junqueira, Pollyana Mendonça de Assis, Nádia Rezende Barbosa Raposo

**Affiliations:** I MD, MSc, PhD. Researcher, Núcleo de Pesquisa e Inovação em Ciências da Saúde (NUPICS), Faculty of Pharmacy, Universidade Federal de Juiz de Fora, Juiz de Fora (MG), Brazil; Physician and Professor, Núcleo de Pesquisa em Dermatologia (NUPEDE), Universidade Federal de Juiz de Fora (UFJF), Juiz de Fora (MG), Brazil.; II MD, MSc, PhD. Physician and Professor, Núcleo de Pesquisa em Dermatologia (NUPEDE), Universidade Federal de Juiz de Fora (UFJF), Juiz de Fora (MG), Brazil.; III PhD. Pharmacist and Professor, Faculty of Pharmacy, Universidade Federal de Juiz de Fora (UFJF), Juiz de Fora (MG), Brazil.; IV MSc. Pharmacist and Doctoral Student, Faculty of Pharmacy, Universidade Federal de Juiz de Fora (UFJF), Juiz de Fora (MG), Brazil.; V MSc. Pharmacist and Doctoral Student, Faculty of Pharmacy, Universidade Federal de Juiz de Fora (UFJF), Juiz de Fora (MG), Brazil.; VI MSc, PhD. Pharmacist and Professor, Faculty of Pharmacy, Universidade Federal de Juiz de Fora (UFJF), Juiz de Fora (MG), Brazil.

**Keywords:** Psoriasis, Arthritis, psoriatic, Retrospective studies, Brazil, Prevalence, Psoriatic arthritis, Plaque psoriasis, Retrospective cross-sectional study, CASPAR, PASI

## Abstract

**BACKGROUND:**

Psoriatic arthritis is the most frequent and impactful comorbidity among psoriatic patients and appears in most cases after skin disease. Dermatologists play a key role in its early diagnosis and treatment.

**OBJECTIVE:**

To determine the prevalence of psoriatic arthritis and associated variables among patients with plaque psoriasis seen at a reference center for treating psoriasis.

**DESIGN AND SETTING:**

Retrospective cross-sectional study conducted among 300 patients at an outpatient clinic in a university center in Juiz de Fora, MG, Brazil.

**METHODS:**

Standardized records of 300 patients with plaque psoriasis were examined. Demographic data and medical variables relating to psoriasis (Psoriasis Area and Severity Index (PASI), family history, age at onset and disease progression) and psoriasis arthritis (CASPAR criteria) were evaluated. Laboratory and radiographic tests in the medical records were reviewed.

**RESULTS:**

Seventy-three (24.3%) of these 300 patients with plaque psoriasis had psoriatic arthritis. Asymmetric oligoarthritis (58.9%) was the most common clinical form, followed by polyarthritis (20.5%), distal interphalangeal arthritis (15.2%) and spondyloarthritis (5.4%). Dactylitis was present in 21.9% and enthesitis in 35.6% of patients. Compared with patients without arthritis, patients with arthritis had higher average age, higher frequency of positive family history of psoriasis, longer duration of evolution and higher PASI rates.

**CONCLUSION:**

Psoriatic arthritis is often underdiagnosed. Since dermatologists perform the initial approach, these professionals need to be trained to diagnose this comorbidity and treat it, together with rheumatologists.

## INTRODUCTION

Psoriasis is a chronic inflammatory pathological condition of recurrent nature and multifactorial etiology that affects about 2% to 5% of people worldwide.^1^^,^^2^ Currently, psoriasis is considered to be a systemic disease and it may be associated with several comorbidities, including psoriatic arthritis.^3-6^ Psoriatic arthritis is a progressive disease, and some patients can progress to severe forms with joint damage and permanent functional changes.^5–8^ Although it was previously recognized as a rare condition, its prevalence among psoriatic patients was found to be high in a recent systematic review, ranging from 4.2% to 33.6%.^9^ In addition, it is considered to be the comorbidity that has the greatest impact on the quality of life of these patients, thus requiring early diagnosis and treatment.^10^

In Brazil, the prevalence of psoriatic arthritis exclusively in patients with plaque psoriasis has not yet been defined. Given this context, and combined with the fact that skin lesions precede joint injuries in more than 80% of cases,^7^^–^^9^ dermatologists have the opportunity to identify patients at risk and diagnose and treat them early.

## OBJECTIVE

The aim of this study was to determine the prevalence of psoriatic arthritis and associated variables among patients who had been diagnosed with plaque psoriasis at a teaching center in Juiz de Fora, MG, Brazil.

## METHODS

### Sample selection and ethics compliance

We conducted a cross-sectional, comparative, retrospective study that included 300 patients with plaque psoriasis who were treated in the psoriasis outpatient clinics of the Dermatology Service at the University Hospital of the Faculty of Medicine, Universidade Federal de Juiz de Fora (UFJF), between January and December 2016. The inclusion criteria were that the patients could be of either sexes, aged between 18 and 60 years, and needed to have a clinical and/or histopathological diagnosis of plaque psoriasis. This diagnosis was made in accordance with the Classification criteria for Psoriatic Arthritis (CASPAR).^11^ Patients with other clinical forms of psoriasis, and those for whom data were missing from the standardized medical records for psoriasis, were excluded. Data collection only started after approval of the investigation by our institution’s ethics committee (protocol 3.142.153; approved on November 2, 2019, by the Research Ethics Committee of the University Hospital, UFJF). All procedures involved in this study were in conformity with the Declaration of Helsinki of 1975, as updated in 2013.

### Clinical, laboratory and radiographic evaluation

The standardized medical records for each patient were reviewed and the following variables were evaluated: sex, age, family history of psoriasis, age at disease onset, duration of the disease, presence of psoriatic arthritis (according to the CASPAR criteria)^11^ and disease severity according to the Psoriasis Area and Severity Index (PASI).^12^ Using PASI, the severity of psoriasis was stratified as mild (PASI < 10) or moderate-to-severe (PASI > 10). Rheumatoid factor and radiographic reports were also reviewed.

### Statistical analyses

Descriptive data analysis was performed, and we assessed normality of distribution by applying the Shapiro-Wilk test. The homogeneity of the variance was assessed by applying the Levene test. When the assumptions of normal distribution and homogeneity of variance were met, the t test was used to ascertain the differences in quantitative variables between the two groups. The chi-square test (χ^2^), or Fisher’s exact test for less than five data points, was used to test for possible differences in the proportions of qualitative variables. In all statistical analyses, the significance level adopted was 5% (P < 0.05). The analyses were performed using the R software package for Windows [R Core Team (2019); version 3.4.4 (R Foundation for Statistical Computing, Vienna, Austria); URL https://www.R-project.org/].

## RESULTS

The characteristics of the patients with plaque psoriasis with and without arthritis are shown in Table 1. Three hundred patients with plaque psoriasis were assessed, among whom 227 (75.7%) only presented skin lesions, while 73 (24.3%) patients had concomitant arthritis. The arthritis patients had a higher average age (49.98 ± 11.12 versus 46.34 ± 13.21 years; P = 0.021).

**Table 1:** General characteristics of psoriatic patients with and without arthritis

Variables	Psoriasis without arthritis n(%) 227 (75.67)	Psoriasis with arthritis n(%) 73 (24.33)	P-value
**Age in years (mean ± SD)**	46.34 ± 13.21	49.98 ± 11.12	0.021
**Male/female (n)**	121/106	40/33	-
**Prevalence of men (%)**	53.3	54.8	0.824
**Positive family history [n(%)]**	69 (30.4)	42 (57.5)	0.001^**^
**Age at diagnosis in years (mean ± SD)**	34.46 ± 15.58	34.60 ± 16.10	0.949
**Duration of psoriasis in years (mean ± SD)**	11.85 ± 11.12	15.63 ± 12.43	0.002^*^
**PASI (mean ± SD)**	12.79 ± 6.75	17.08 ± 4.68	0.001^**^
**Severity [n(%)]**			0.001^**^
Mild	58 (25.5)	1 (1.4)	-
Moderate-to-severe	169 (74.5)	72 (98.6)	-

PASI = Psoriasis Area and Severity Index; SD = standard deviation. ^*^P < 0.01; ^**^P < 0.001.

**Figure 1:**
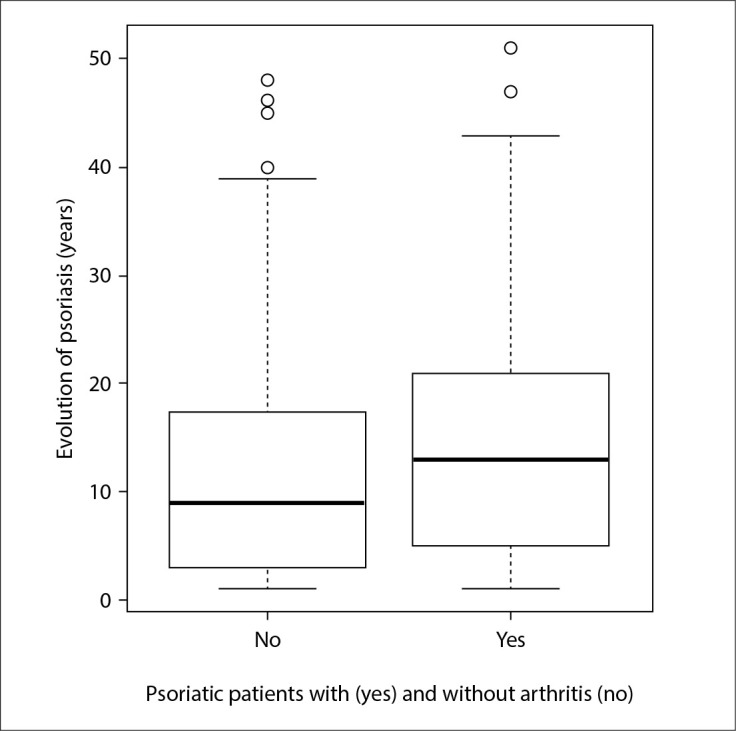
Comparison of duration of psoriasis between patients with and without psoriatic arthritis. The disease duration was longer in the arthritis group (15.63 ± 12.43 versus 11.85 ± 11.12 years; P < 0.01).

The distribution according to sex was similar in the two groups, as also was the age at diagnosis of the disease, which started on average at 34 years of age (patients with arthritis: 34.60 ± 16.10 years, versus patients without arthritis: 34.46 ± 15.58 years, P = 0.949). A positive family history was statistically more frequent among patients with arthritis (57.5% versus 30.4%, P < 0.001), and the disease duration was longer in the arthritis group (15.63 ± 12.43 versus 11.85 ± 11.12 years; P < 0.01) (Figure 1).

**Figure 2:**
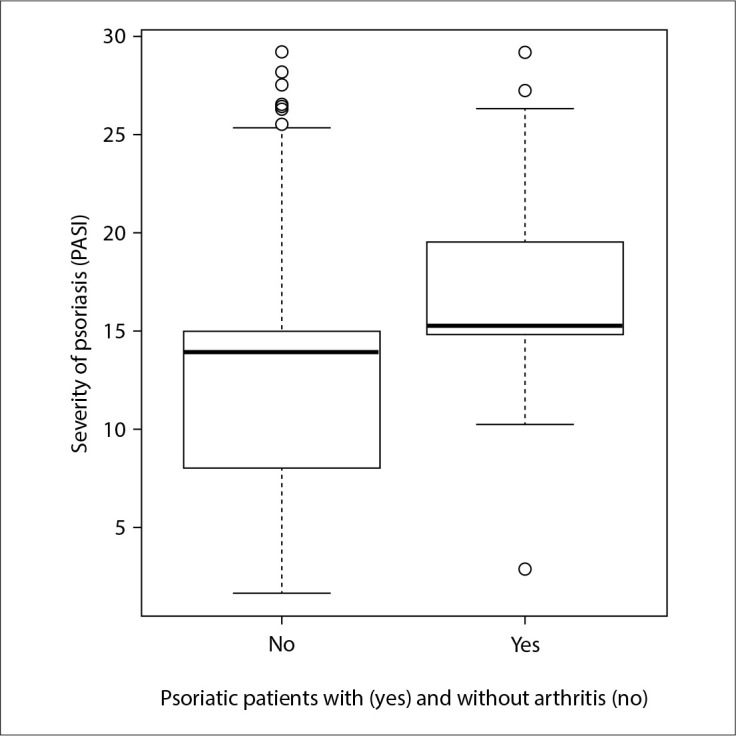
Comparison of Psoriasis Area and Severity Index (PASI) between patients with and without psoriatic arthritis. PASI values were significantly higher in the arthritis group (17.08 ± 4.68 versus 12.79 ± 6.75; P < 0.001).

More than 80% of the patients in the study had moderate-to-severe psoriasis, and PASI values were significantly higher in the arthritis group (17.08 ± 4.68 versus 12.79 ± 6.75; P < 0.001) (Figure 2).

**Table 2:** Clinical patterns of psoriatic arthritis (n = 73)

Clinical manifestations	n(%)
Asymmetric oligoarthritis	43 (58.9)
Polyarthritis	15 (20.5)
Distal interphalangeal arthritis	11 (15.2)
Spondyloarthritis	4 (5.4)
Dactylitis	16 (21.9)
Enthesitis	26 (35.6)

Regarding joint patterns, the most common clinical form was asymmetric oligoarthritis (58.9%), followed by polyarthritis (20.5%), distal interphalangeal arthritis (15.2%) and spondyloarthritis (5.4%). Dactylitis was present in 21.9% of patients and enthesitis in 35.6% (Table 2).

## DISCUSSION

This study investigated the prevalence of psoriatic arthritis among 300 patients with plaque psoriasis who were treated by a dermatologist at a teaching center that is a reference center for treating psoriasis, in Juiz de Fora, Brazil. The medical care included use of a standardized questionnaire for psoriasis, and joint involvement was analyzed through the CASPAR criteria, which has high specificity (98.7%) and sensitivity (91.4%) for early diagnosis of psoriatic arthritis.^11^^,^^13^ These criteria are also used for dispensation of high-cost drugs by the Brazilian government.

The prevalence rate found in the present study was 24.33%. In Brazil, previous studies using the CASPAR criteria have shown prevalences of 35%^14^ and 33%.^15^ However, one of the reasons for these differences is probably the fact that the authors of the previous studies included patients with other clinical forms of psoriasis in the assessment, accounting for 17.59%^14^ and 20.41%^15^ of their total samples.

Similar results were observed by Christophers et al.,^16^ who evaluated 1,560 patients in Europe using the CASPAR criteria and estimated the prevalence at 20.5%. Reich et al.^17^ evaluated the prevalence and clinical pattern of psoriatic arthritis among 1,511 patients with plaque psoriasis: 312 (20.6%) had psoriatic arthritis, and 85% of these cases were diagnosed for the first time through that study. In contrast, an American study^18^ conducted by means of telephone interview found an 11% prevalence. These differences between studies are explainable in terms of geography, ethnicity, genetic background, clinical forms and different diagnostic criteria used to define psoriatic arthritis.^9^ In approximately 80% of the cases, the skin disease precedes the joint disease by at least 10 years. However, in a minority of cases (10%-15%), arthritis may precede cutaneous involvement.^7^^,^^11^

In Brazil, access to a rheumatologist is limited, even in university centers. However, a well-trained and qualified dermatologist can assess skin and joints. Early detection of arthritis is a key issue and gives dermatologists an important role in detection and management of these patients. Among the characteristics of patients with concomitant arthritis, the following were relevant: older age, greater frequency of a positive family history, longer evolution time and higher PASI.

The onset of psoriasis had no relationship with sex or age, in agreement with previously reported data.^19^ However, the age at onset of psoriasis has been reported by other authors^17^ to be a predictor of psoriasis arthritis. Other factors associated with arthritis risk include the following: several markers of psoriasis severity, such as higher PASI,^5^^,^^20^ body surface area (BSA)^17^^,^^19^ and Dermatology Life Quality Index (DLQI);^17^^,^^21^ greater lengths of hospitalization due to psoriasis in the last five years; more workdays lost in the last 12 months;^17^ and having more than three body sites affected by psoriasis.^5^

In our study, patients with psoriatic arthritis had higher PASI values than those without arthritis, in agreement with a previous study,^17^ thus showing that a severe skin condition is associated with a risk of psoriatic arthritis. Although we did not evaluate the association between skin severity and the number of joints involved, a previous study showed that the correlation between the skin and the joints may be low: a patient may have mild psoriasis and severe arthritis, and the opposite is also true.^22^ Regarding the clinical forms presented by our patients, oligoarthritis was the most common, and axial involvement was the least, in agreement with previous reports.^23^ The data relating to the presence of enthesitis (35.6%) and dactylitis (21.9%) were in accordance with previous studies,^24^^,^^25^ which stated that these rates can reach up to 50% among patients, and that presence of dactylitis is related to earlier joint damage, such as bone neoformation and erosion. To our knowledge, our study was the first in Brazil to assess the prevalence of psoriatic arthritis among patients with plaque psoriasis, in which dermatologists made the diagnosis by using the CASPAR criteria. Therefore, the data in this study can be considered representative of a considerable proportion of patients with psoriasis in Brazil.

According to our findings, dermatologists can expect that out of every 10 patients with plaque psoriasis, about 2.5 of them will have psoriatic arthritis. In addition, the severity of the disease verified through PASI among patients with concomitant arthritis emphasizes that there is a need for dermatologists to become familiar with the diagnostic criteria and clinical findings, which are already well documented for arthritis. Increased PASI and involvement of the scalp, nail and intergluteal or perianal groove form clinical markers that facilitate early diagnosis and treatment.

## CONCLUSION

Psoriatic arthritis is often underdiagnosed. Since dermatologists perform the initial approach, these professionals need to be trained to diagnose this comorbidity and treat it, together with rheumatologists.
